# Comparison of latissimus dorsi tendon transfer with subscapularis release versus sliding of internal rotation contracture in obstetrical brachial plexus paralysis sequela

**DOI:** 10.1186/s13018-022-03065-w

**Published:** 2022-03-15

**Authors:** Ashraf M. Abdelaziz, Mohammed A. AbdAlfattah, Faisal Ahmed Hashem El-sherief, Yaser El Sayed Hassan Wahd, Hany Abdel Gawwad Soliman, Hassan Fathy El Behairy, Mahmoud Ali Ismail

**Affiliations:** Alzhraa University Hospital, Faculty of Medicine for Girls, Al-Azhar University, Cairo, 11517 Egypt

**Keywords:** Subscapularis, Brachial plexus, Internal rotation contracture, Erb’s palsy

## Abstract

**Background:**

The purpose of this study was to compare the functional results of latissimus dorsi (LD) tendon transfer with those of subscapularis (SS) muscle release versus sliding.

**Methods:**

Fifty-six patients with internal rotation contracture and external rotation (ER) weakness as sequelae of Erb’s palsy were included in the study. Of the patients, 24 were included in group 1 (11 boys and 13 girls), with a mean age of 2 years 8 months (range 1.5–5 years) and a follow-up period of 62 months (range 38–68 months). The patients in group 1 underwent LD tendon transfer, with internal rotation contracture and SS release procedures. Thirty-two patients were included in group 2 (18 boys and 14 girls), with a mean age of 2 years 6 months (range 1.5–4.8 years) and a follow-up period of 58 months (range 38–68 months). The patients in group 2 underwent LD tendon transfer with SS sliding.

**Results:**

A significant improvement in preoperative passive ER from − 3.6° to 67.3° after operation was observed in group 1. In group 2, preoperative passive ER in adduction improved from 0° to 72.3°. We found no significant difference (*P* = 0.1) in postoperative improvement in active ER in both groups (group 1 vs. group 2: 75° vs. 77.3°). Similarly, no significant difference (*P* = 0.7) in postoperative improvement in passive ER was found between the groups (group 1 vs. group 2: 71° vs. 72.3°).

**Conclusions:**

LD tendon transfer with SS release or sliding is an effective procedure to improve shoulder ER in patients with OBPP, with no inferiority of SS muscle release or sliding for internal rotation contractures and increased passive range of shoulder motion.

**Level of evidence:**

Level III; Retrospective Cohort Comparison; Treatment Study.

## Background

Internal rotation contracture is a common sequela of obstetric brachial plexus palsy (OBPP) and is an active external rotation (ER) deficit. Failure of the muscle to recover can cause glenohumeral joint deformity, shoulder joint subluxation, or shoulder dislocation [1, 2].

For patients with OBPP, internal rotation contractures, together with weakening of the external rotators that leads to progressive bony deformities due to muscle imbalance and shortening of the dominant muscles of the shoulder, including the pectoralis major (PM) and subscapularis (SS), may cause secondary changes such as glenoid hypoplasia and flattening of the humeral head, resulting in posterior subluxation [3–6].

Release or tenotomy of the SS muscle or sliding of the SS muscle on the anterior face of the scapula [7] can be associated with either transfer of the teres major (TM) and latissimus dorsi (LD) muscles to the greater tuberosity [8] or fixation of the transferred tendons to the rotator cuff or infraspinatus muscle, thereby improving the stabilizing effect of the rotator cuff by yielding greater glenohumeral abduction [8, 9].

Several authors [10, 11] advice release of SS without capsulotomy and reported good results. Sliding of SS was initially described by Carlioz [11] and Brahimi and subsequently advocated by Gilbert et al. [12] The proposed benefits of this procedure include the ability to achieve intraoperative correction of the contracture while avoiding the potential pitfalls of anterior releases, such as instability and loss of internal rotation strength [13].

This study aimed to compare the functional results of LD tendon transfer with SS release with those of muscle sliding.

## Materials and methods

In this prospective cohort study conducted between 2013 and 2019, 56 patients with internal rotation contracture and ER weakness as sequelae of Erb’s palsy who underwent surgery at our hospital were included. The inclusion criteria were age ≤ 5 years, no subluxation or dislocation, and no history of previous surgery. The study excluded patients aged > 5 years, who had lower trunk palsies, transfers without releases, treatment with derotational osteotomies, flail shoulders, arthritic shoulder joint, and previous neurotization. Patients who could undergo follow-up were selected.

The study population comprised 56 patients. There were 24 patients in group 1 (11 boys and 13 girls), with a mean age of 2 years 8 months (range 1.5–5 years). Of these patients, 14 (58%) were right-handed. The mean follow-up period was 62 months (range 38–68 months). The patients in group 1 underwent LD tendon transfer together with internal rotation contracture release and SS release (PM tendon Z-plasty in two cases). There were 32 patients in group 2 (18 boys and 14 girls), with a mean age of 2 years 6 months (range 1.5–4.8 years), and 20 (62%) were right-handed. The mean follow-up period was 58 months (range 38–68 months; Table 1). The patients in group 2 underwent LD tendon transfer and SS sliding. No contraindication of tendon transfer, particularly passive motion with scapular stabilization, was found in the supple joint. Computed tomography and magnetic resonance imaging were not conducted in this study. Randomization was performed by sequential selection.

**Table 1:** Patient demographics

Characteristic	Group 1 (24)	Group 2 (32)
Age, mean (range)	2 years and 8 months (1.5–5 years)	2 years and 6 months (1.5–4.8 years)
Male sex	11 (46%)	18 (56%)
Female sex	13 (54%)	14 (44%)
Right-handed	14 (58%)	20 (62%)
Left-handed	10 (42%)	12 (38%)

Written consent was obtained from all the patients, and the study was performed in accordance with the principles of the Declaration of Helsinki.

A complete follow-up was conducted for all the patients by the main surgeons, with postoperative clinical evaluation at 1.5, 2, 4, 6, and 12 months and every 6 months thereafter at the outpatient clinic. The patients underwent the following evaluations preoperatively and postoperatively: 1) passive ER during abduction and adduction; 2) presence of abduction contracture, ER contracture, and active global abduction; and 3) active ER. A modified Mallet score [11] was used to classify these conditions (Table 2).

**Table 2:** Passive external rotation during adduction

External rotation	Group 1 (24) Number (%)	Group 2 (32) Number (%)
Less than neutral	8 (33.5)	8 (25)
None to neutral (up to 0°)	10 (41.5)	16 (50)
Passive external rotation (> 0–30°)	6 (25)	8 (25)
Passive external rotation (30–60°)	0 (0)	0 (0)
Passive external rotation (30–90°)	0 (0)	0 (0)

### Surgical technique

#### Group 1

**Fig. 1:**
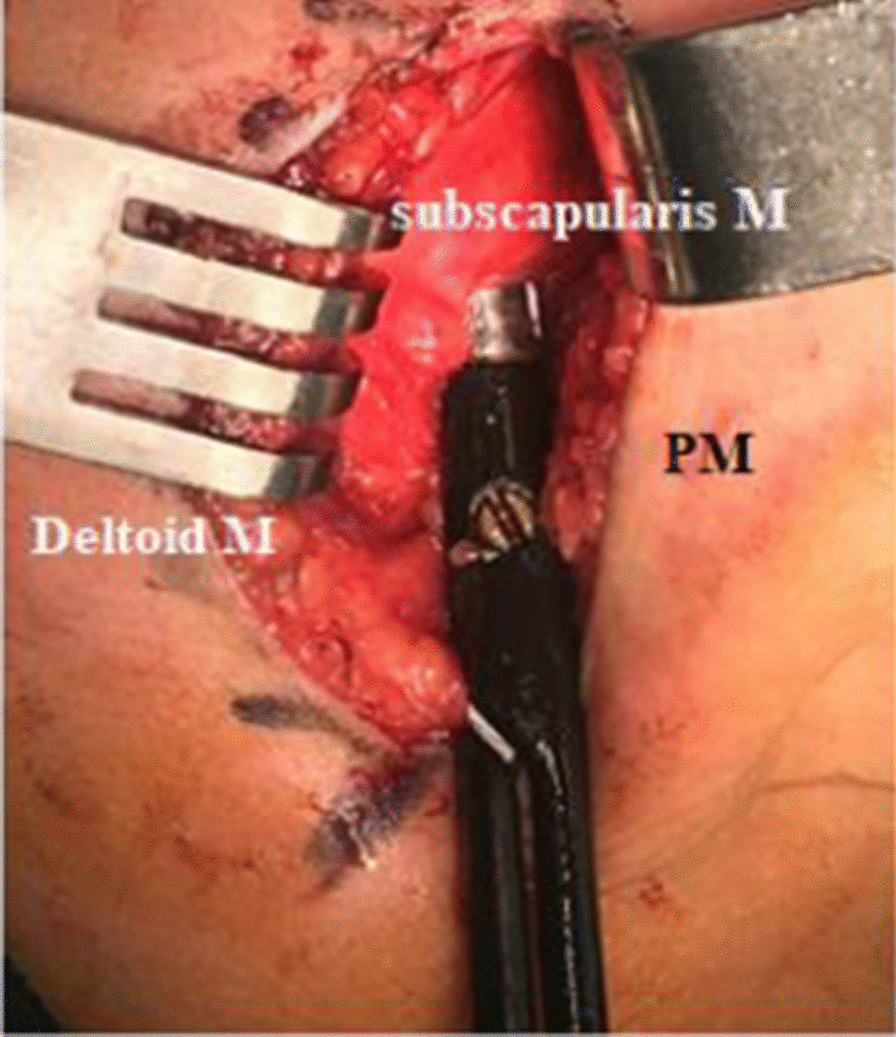
Deltopectoral approach to address anterior rotation in group 1, identification of subscapularis tendon and release. *PM* pectoralis major

The patient was placed in the lateral decubitus position with the affected side up and the arm held next to the side. The anterior deltopectoral approach was used to release the soft tissue [5, 9] (Fig. 1).

The SS tendon was identified and released completely starting from the upper border because it was easier to release from that point. ER was applied to the arm as it enables better stretching of the SS and easier dissection. The capsule was protected. Z-plasty of the PM tendon was performed in two cases with severe internal rotation contructure, and ER was tested in adduction.

#### Group 2

**Fig. 2:**
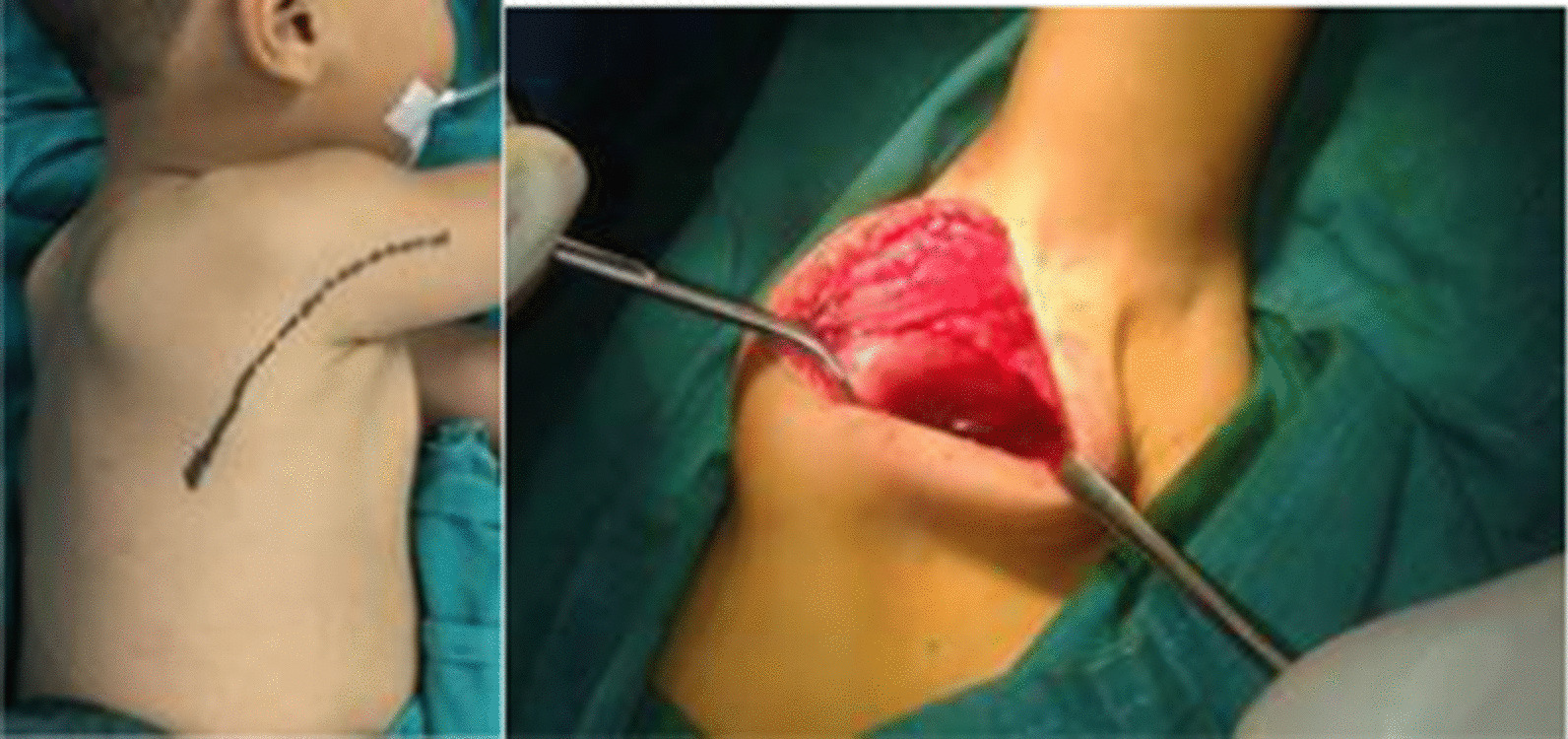
Posterior subscapularis sliding in group 2. **A** Curved incision for posterior approach, **B** subscapularis sliding

A curved wide C-shaped incision was made along the posterior axillary fold. Then, the fascia of the LD was identified. The anterior border of the LD was retracted posteriorly, and deeper dissection was performed to expose the anterior surface of the scapula and SS muscles. The inferior angle of the scapula was held with a towel clip. Sliding of SS muscle was performed using a periosteal elevator, and passive ER was assessed after sliding (Fig. 2).

The LD muscle was transferred in both groups to the rotator cuff (to the infraspinatus tendon). Tension of the transferred tendon should keep the arm slightly abducted and externally rotated.

Follow-up was conducted 1.5 months postoperatively for cast removal, and physiotherapy was indicated in the form of abduction, ER exercise, passive ER during adduction, and passive internal rotation. The patient’s family was instructed how to perform the daily therapy at home.

At each visit, we measured and recorded the patient’s active shoulder abduction, passive ER with adduction, active ER, ER contracture grade, and modified Mallet score [5].

### Statistical analysis

The data were coded and entered using SPSS version 23 statistical package (Statistical Package for the Social Sciences). The data were summarized as quantitative values and as frequency (count) and relative frequency (percentage) for categorical variables. The quantitative variables were compared, and differences with *P* values of < 0.05 were considered statistically significant.

## Results

Marked improvements in preoperative shoulder abduction from 63.3° to 157°, active ER in abduction from 7.7° to 82.7°, and preoperative passive ER in adduction from − 3.6° to 67.3° were observed postoperatively in group 1, with significant differences (*P* < 0.001).

In group 2, preoperative active ER in abduction improved from 7.7° to 85° postoperatively. Preoperative shoulder abduction improved from 67.3° to 158.3° postoperatively. A marked improvement in preoperative passive ER in adduction from 0° to 72.3° was also observed postoperatively, with a significant difference (*P* < 0.001).

We found no significant difference (*P* = 0.4) in postoperative improvement in shoulder abduction in both groups (group 1 vs. group 2: 94° vs. 91°). No significant difference (*P* = 0.1) in postoperative improvement in active ER was found between the two groups (group 1 vs. group 2: 75° vs. 77.3°). In addition, no significant difference (*P* = 0.7) in postoperative improvement in passive ER was found between the groups (group 1 vs. group 2: 71 vs. 72.3; Table 3).

**Table 3:** Comparative analysis of the postoperative results between the two groups

	Mean (°)	SD (°)	*t* value	*P* value
*Postoperative abduction*				
Group 1	94°	9.8	0.7	0.4
Group 2	91°	12.2		
*Active external rotation*				
Group 1	75°	4.2	1.1	0.1
Group 2	77.3°	6.5		
*Passive external rotation*				
Group 1	71°	11.8	0.3	0.7
Group 2	72.3°	11.4		

*P* values more than 0.05 were considered as statistically not significant

Loss of the last degrees of internal rotation was observed in two patients in group 1 at the early follow-up (4 mon) when the patients were asked to place their hands on their abdomens. Exercises and physiotherapy performed by holding the scapula against the ribs while flexing and internally rotating the arm were found to be generally useful. No infection or subluxation was recorded in both the groups.

## Discussion

Internal rotation contracture in OBPP usually occurs because of muscle imbalance (strong internal rotator against the external rotators) around the shoulder and leads to weak shoulder abduction and ER, causing limitations in performing daily activities that require the use of the hands above the shoulder level [14].

Various treatment options have been suggested, including LD tendon transfer, anterior open SS release or SS muscle sliding, full or partial release of the SS muscle, and humerus derotational osteotomies [1, 2, 5, 9, 15]. In this study, we performed a functional comparison between internal rotation anterior open SS release and SS muscle sliding. The treatment goals in OBPP sequelae are to eliminate internal rotation contracture and improve active ER. To determine the best surgical option to manage internal rotation contracture and restore ER, we performed anterior open SS release and posterior SS muscle sliding with LD tendon transfer.

In this study, we performed surgery in patients aged 1.5–5 years who had no advanced glenohumeral deformities to provide a chance for growth-driven joint remodeling, as indicated in the literature [1, 2, 5, 9]. These studies have suggested that such children should undergo surgery as early as possible to avoid worsening of glenohumeral deformation, which leads to severe internal rotation contractures, resulting in poor surgical outcomes. Better results are expected in younger children.

To date, no studies have compared the effectiveness of anterior open release with that of muscle sliding for the improvement in internal rotation contraction. Most studies included case series of surgical techniques. Anterior open SS release with transfers of the TM and LD tendons to the posterior aspect of the humerus was first described by L’Episcopo in 1934. Later, Hoffer et al. [8] modified this technique and proposed that the LD and TM tendons should be transferred to the rotator cuff without SS release. El-Gammal et al. [5] performed open SS tendon release with or without open PM tendon lengthening in 109 patients. Sever et al. [13], Cohen et al. [7], Aydin et al. [16], and Terzis et al. [15] performed tendon transfers with open SS release with or without PM tendon release, which showed clinical and functional improvements. In the present study, we performed anterior release with PM Z-plasty in two patients and reported significant improvements in passive and active ERs.

It has the advantage of achieving full intraoperative correction of the contracture while avoiding the pitfalls of anterior release, safely achieving the goal of passive ER restoration and avoiding overlengthening and weakening of the SS [17–19].

Kambhampati [20] observed a 10° loss of internal rotation after SS sliding but a higher loss in internal rotation of up to 42° after arthroscopic tenotomy of the SS tendon. We observed a loss of internal rotation of 10° and hypothesized that this occurred because of a loss of internal rotation power and stronger ER. With follow-up and physiotherapy, this internal rotation limitation improved.

Waters [2] and Pearl [18] observed LD tendon transfers to the rotator cuff with glenohumeral joint reduction and remodeling of glenohumeral dysplasia in most patients and a subsequent improvement in ER at all ages, but the most impressive reorganization has been reported to occur in younger children.

In the present study, we compared open SS release plus with SS posterior sliding and found no significant differences in postoperative abduction and degrees of active or passive ER. For anterior release, two approaches are needed, which are easy to perform for experienced surgeons. The limitations of this study are the lack of radiographic findings and the limited number of patients in comparison with the high incidence of bony deformities.

## Conclusion

Without inferiority to SS muscle release or sliding, LD tendon transfer with those of subscapularis (SS) muscle release or sliding is an effective procedure for internal rotation contractures as well as improvement in shoulder ER in patients with obstetrical brachial plexus palsy, except anterior release need another anterior approach.

## Abbreviations

PM: Pectoralis major
LD: Latissimus dorsi
ER: External rotation
SS: Subscapularis
OBPP: Obstetric brachial plexus palsy
